# Supplementary material for: Extracellular Expression of Feruloyl Esterase and Xylanase in Escherichia coli for Ferulic Acid Production from Agricultural Residues
Source: Microorganisms. 2023 Jul 25;11(8):1869. doi: 10.3390/microorganisms11081869 (PMC10456899; doi:10.3390/microorganisms11081869)
Supplement: Supplementary file 1 [file microorganisms-11-01869-s001.zip › microorganisms-2469497-supplementary.pdf]

Table S1 Primers used in this study

Primers	Sequence（5′-3′）
F-*Eco*R I-*fae*	CATGAATTCGGCTCGTCTGCAG
R-*Hin*d III-*fae*	GCTAAGCTTTTAACCGAACATCC
F- *Eco*R I-*xyn*	CACGAATTCGGCTACCACCATCACCGAAAAC
R-*Hin*d III-*xyn*	GCTAAGCTTACCAGAGTTAGAACCAGCACCAG
F1-*Bgl* II-*fae*	GGTAACGACAACGTTAAAAGATCTGCTCGTCTGCAG
F1-2	GAAGACAACTTCAAACACCTGCTGGGTAACGACAAC
F1-*Nde* I	GCTCATATGGAAGGTAACACCCGTGAAGACAACTTC
F2-*Bgl* II-*fae*	GGTGACCGTGAAGAACCGAGATCTGCTCGTCTGCAG
F2-2	GAACGTGACGGTCTGACCCTGGTTGGTGACCGTGAAG
F2-*Nde* I	GCTCATATGTCTCGTATCACCATCGAACGTGACGGTC
F3-*Nde* I	GCTCATATGTCTCGTGTTACCATCGAACGTGACGGTC
F4-2	GAACGTGACTCTCTGACCCTGGTTGGTGACCGTGAAG
F4-*Nde* I	GCTCATATGTCTCGTATCACCATCGAACGTGACTCTC
F5-*Bgl* II-*fae*	GTCTGCTGGAAGGTACCAGATCTGCTCGTCTGCAG
F5-2	ACGTGACGGTCTGAAACTGTACGGTCTGCTGGAAGG
F5-*Nde* I	GCTCATATGGAAATCACCATCAAACGTGACGGTCTG
R-*Xho* I-*fae*	CATCTCGAGACCGAACATCCATTCAACAACTTTAGC
F-*Bgl* II-*xyn*	CACAGATCTGCTACCACCATCACCGAAAAC
R-*Xho* I-*xyn*	CATCTCGAGACCAGAGTTAGAACCAGCACCAG
F-*Nde* I-*osmY*	GCTCATATGACTATGACAAGACTGAAGATTTCG
R-*osmY*-3GS	AGAACCAGAACCAGAACCCTTAGTTTTCAGATCA
F-3GS-*fae*	GGTTCTGGTTCTGGTTCTGCTCGTCTGCAGATC
F-3GS-*xyn*	GGTTCTGGTTCTGGTTCTGCTACCACCATCACCG
F1-*Nco* I	CATCCATGGAAGGTAACACCCG
R-*Eco*R I-*fae*	GCTGAATTCTTAACCGAACATCC
